# Dental caries and associated factors among Chinese children and adolescents: A protocol for systematic review and meta-analysis

**DOI:** 10.1097/MD.0000000000025829

**Published:** 2021-05-07

**Authors:** Zhenxian Huang, Meixuan Su, Qiaojing Wang, Wenjie Li, Haimin Jiang

**Affiliations:** a Xiamen University Zhongshan Hospital; b Xiamen Haicang Hospital, Xiamen, Fujian province, China.

**Keywords:** adolescents, children, China, dental caries, meta-analysis, protocol, risk factors

## Abstract

**Background:**

Dental caries is a common disease under the action of many factors. Dental caries may occur in all age groups, among which children and adolescents are at high risk of dental caries. Early identification of the risk factors of dental caries is very important for clinical staff to prevent and intervene as soon as possible and reduce the incidence of dental caries. Although Chinese scholars have studied and summarized the risk factors of dental caries in children and adolescents, the conclusions are different. Therefore, in this study, meta-analysis was used to summarize the risk factors of dental caries in Chinese children and adolescents, and to explore the characteristics of high-risk groups of dental caries, so as to provide reference for early detection and prevention of dental caries.

**Methods:**

Medical specialty databases like PubMed, Embase, Cochrane Library, Web of Science, China Biology Medicine Database, China National Knowledge Infrastructure, China Science and Technology Journal Database, and Wanfang Database were consistently and exhaustively searched. According to the method of evidence-based medicine, the inclusion and exclusion criteria were established, and the meta-analysis of all eligible research results was carried out by using Review Manager 5.3 software.

**Results:**

We will disseminate the findings of this systematic review and meta-analysis via publications in peer-reviewed journals.

**Conclusions:**

Our study was carried out to estimate the pooled prevalence of dental caries and its associated factors among Chinese children and adolescents.

**OSF Registration Number:**

DOI 10.17605/OSF.IO/RA9D6.

## 1 Introduction

Dental caries is a disease that is chronic undergoing disruption of teeth under multiple factors dominated by bacteria.^[1–3]^ And it is a global public health problem and the most common non-communicable disease.^[4]^ In developed countries, the cost of treating this problem is higher, accounting for 5% to 10% of the health care budget, and it is also a common cause of hospitalization.^[5]^ The incidence of dental caries is increasing due to the unrestricted use of sugary foods, bad oral care habits, and underutilization of medical services.^[6–8]^

In recent years, due to changes in dietary structure, the incidence of dental caries in China has shown a significant upward trend. For example, the incidence of dental caries in 3-year-old children has reached 50.8%, and the incidence of dental caries in 5-year-old children has reached 71.9%.^[9]^ This suggests that dental caries has become a common and high incidence of oral diseases in minors, which has a certain impact on the health, growth, and development of minors.

Although many studies on dental caries between different subjects have been carried out in China, their results are inconsistent.^[10–21]^ Therefore, this systematic review and meta-analysis will further explore the total prevalence and related factors of dental caries in Chinese children and adolescents, so as to provide evidence-based medicine for the prevention and treatment of missing teeth. Understanding the total prevalence and influencing factors of dental caries in Chinese children and adolescents will help to take appropriate measures to control the problem.

## 2 Methods

### 2.1 Study registration

This protocol has been registered on Open Science Framework (registration number: DOI 10.17605/OSF.IO/RA9D6). This report is based on the preferred reporting items for systematic review and meta-analysis protocols.^[22]^

### 2.2 Eligibility criteria

Inclusion criteria:

(1) Study design: We will include case-control study, cohort study, and cross-sectional study; the study involves the risk factors of dental caries.
(2) Participants: People aged between 0 and 17 years old will be included.
(3) Outcomes: The results of the study are consist of the specific values of odds ratio (OR) and 95% confidence interval (95% CI) of risk factors.

Exclusion criteria:

(1) The full text cannot be obtained normally or the extracted data will be affected;
(2) Repeatedly published literatures;
(3) Review, systematic review, conference, animal experiments, and other literatures.

### 2.3 Search strategy

Studies on dental caries in China were consistently and exhaustively searched using medical specialty databases of PubMed, Embase, Cochrane Library, Web of Science, China Biology Medicine Database, China National Knowledge Infrastructure, China Science and Technology Journal Database, and Wanfang Database. The retrieval time was from the establishment of the database to March 2021. The search terms are “Dental caries,” “China,” “Children,” “Adolescents,” “Risk factor,” “Risk assessment,” “Multivariate analysis,” and “Multivariable logistic regression,” etc. And these search terms are summarized in Table 1.

**Table 1:** Search strategy used in PubMed database.

Number	Search terms
#1	Dental Caries[MeSH]
#2	Caries, Dental[Title/Abstract]
#3	Dental Decay[Title/Abstract]
#4	Dental White Spots[Title/Abstract]
#5	White Spots[Title/Abstract]
#6	Decay, Dental[Title/Abstract]
#7	Dental White Spot[Title/Abstract]
#8	White Spot, Dental[Title/Abstract]
#9	White Spots, Dental[Title/Abstract]
#10	Spot, White[Title/Abstract]
#11	Spots, White[Title/Abstract]
#12	White Spot[Title/Abstract]
#13	or/1–12
#14	Child[MeSH]
#15	Children[Title/Abstract]
#16	Adolescent[MeSH]
#17	Adolescence[Title/Abstract]
#18	Youth[Title/Abstract]
#19	Adolescents[Title/Abstract]
#20	Adolescents, Female[Title/Abstract]
#21	Adolescents, Male[Title/Abstract]
#22	Teenagers[Title/Abstract]
#23	Teens[Title/Abstract]
#24	Adolescent, Female[Title/Abstract]
#25	Adolescent, Male[Title/Abstract]
#26	Female Adolescent[Title/Abstract]
#27	Female Adolescents[Title/Abstract]
#28	Male Adolescent[Title/Abstract]
#29	Male Adolescents[Title/Abstract]
#30	Teen[Title/Abstract]
#31	Teenager[Title/Abstract]
#32	Youths[Title/Abstract]
#33	or/14–32
#34	China[Title/Abstract]
#35	Chinese[Title/Abstract]
#36	or/34–35
#37	Risk factor[Title/Abstract]
#38	Risk assessment[Title/Abstract]
#39	Multivariate analysis[Title/Abstract]
#40	Multivariable logistic regression[Title/Abstract]
#41	or/22–25
#42	#13 and #33 and #36 and #41

### 2.4 Study selection

**Figure 1:**
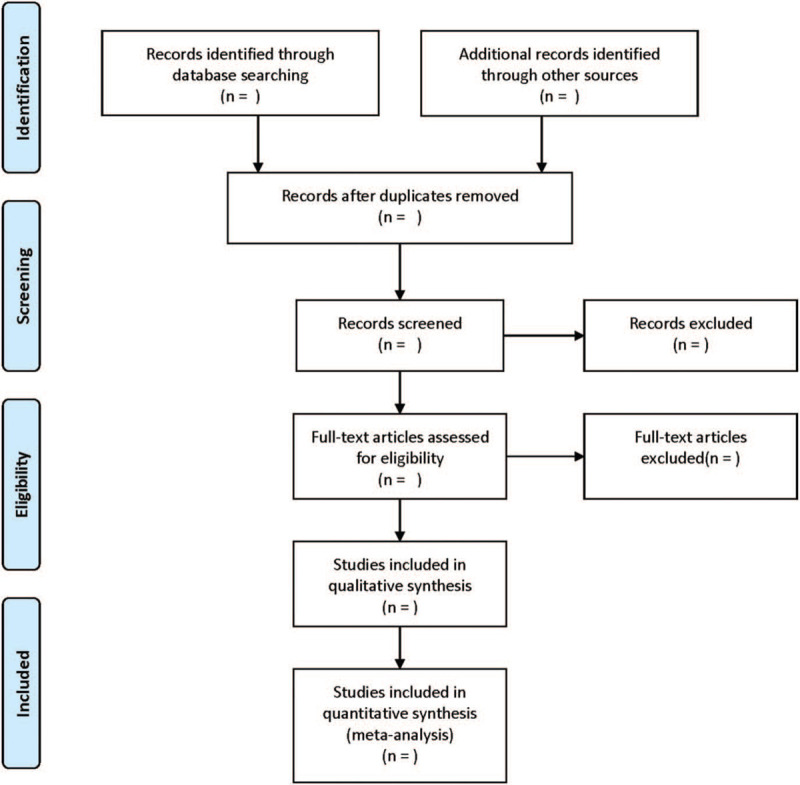
Flow chart of study selection.

Literature was screened by 2 researchers, and a third researcher judged whether to include the literature when having conflict opinions. Firstly read the title and summary. Secondly, the full text is re-screened according to the detailed entries of the literature inclusion criteria. The process of the selection is exhibited in Fig. 1.

### 2.5 Data extraction

The 2 researchers extracted information in a format prepared in an Microsoft Excel spreadsheet. The information entries are as follows: author's name, year of publication, region, study design used, age range, sample size, as well as results of interest, prevalence, and factors associated with dental caries.

### 2.6 Assessment of the risk of bias

Quality of eligible studies was checked by employing Newcastle-Ottawa Scale (NOS) before analysis.^[23]^ NOS scoring ≥6 means that the literature quality is great. Applying Combie cross-sectional research evaluation tool to evaluate the methodological quality of cross-sectional research.

### 2.7 Data analysis

We will use OR and 95% CI to represent. If there are no findings of statistical heterogeneity, the Mantel–Haenszel method fixed effect model would be adopted for data synthesis,^[24]^ and if there is significant statistical heterogeneity, we will apply the DerSimonian–Laird random effect model.^[25]^ All participants will explore the possible causes from clinical and methodological perspective and provide a descriptive or subgroup analysis. The data analysis of this study will be conducted through Review Manager Version 5.3 software.

### 2.8 Assessment of heterogeneity

The heterogeneity included in the results of the study was analyzed by performing the Chi-square test (the test level was α = 0.1) and combined with *I*^2^ to quantitatively determine the size of the heterogeneity. When *P* < .1 or *I*^2^ > 50%, the random effect model will be adopted for the combined analysis. Otherwise, the fixed effect model will be used for the combined analysis.

### 2.9 Subgroup analysis

We set up a subgroup analysis according to the type of the study, age, and region of the participants.

### 2.10 Sensitivity analysis

We can use different models to analyze the same data and eliminate small sample data, and then compare the results, so as to reflect the stability of the research results.

### 2.11 Assessment of reporting biases

The inverted funnel diagram in Review Manager5.3 software takes SE (logOR) as ordinate and OR value as Abscissa, and the figure shows a symmetrical inverted funnel shape without bias, otherwise, there will be a missing corner.

### 2.12 Management of missing data

If there is missing data in the article, please contact the corresponding author or the first author through email to obtain accurate data. If the author has lost the relevant data, or is unable to contact, only carry out descriptive analysis and no meta-analysis.

### 2.13 Ethical review and informed consent of patients

The content of this article does not involve moral approval or ethical review and will be presented in print or at relevant conferences.

## 3 Discussion

The results of foreign studies show that the incidence of dental caries in 4-year-old children ranges from 12% to 98%, while the incidence of deciduous tooth caries in Chinese 5-year-old children reaches 70.9%, which is significantly higher than that of 10 years ago.^[26]^ Dental caries not only have an impact on the health of children, such as causing acute and chronic pain, causing serious infection, resulting in a decline in the quality of life, its high incidence also brings a heavy social burden.^[20,21,27]^ It is reported that in 2010, dental expenses for children under the age of 5 in the United States exceeded $1.55 billion.^[28]^ The results of etiological studies show that the occurrence of dental caries in children is related to host susceptibility, cariogenic flora, and suitable growth environment.^[29–31]^

In recent years, many scholars have devoted themselves to the research on the factors related to dental caries in children and adolescents, but there are problems such as scattered research factors and inconsistent research results, so it is difficult to draw comprehensive and reliable conclusions. This paper makes a meta-analysis of the observational research on this topic published in China recently, in order to obtain more robust results through a large sample of research, and to provide a theoretical basis for higher quality research in the future.

## Author contributions

**Conceptualization:** Zhenxian Huang.

**Data curation:** Zhenxian Huang, Qiaojing Wang.

**Funding acquisition:** Zhenxian Huang.

**Methodology:** Meixuan Su.

**Project administration:** Zhenxian Huang.

**Supervision:** Zhenxian Huang.

**Validation:** Qiaojing Wang, Haimin Jiang.

**Visualization:** Qiaojing Wang, Wenjie Li.

**Writing – original draft:** Zhenxian Huang, Meixuan Su.

**Writing – review & editing:** Zhenxian Huang, Meixuan Su.
